# Life Cycle and Morphology of a Cambrian Stem-Lineage Loriciferan 

**DOI:** 10.1371/journal.pone.0073583

**Published:** 2013-08-09

**Authors:** John S. Peel, Martin Stein, Reinhardt Møbjerg Kristensen

**Affiliations:** 1 Department of Earth Sciences (Palaeobiology), Uppsala University, Uppsala, Sweden; 2 Natural History Museum of Denmark, University of Copenhagen, Copenhagen, Denmark; University of Birmingham, United Kingdom

## Abstract

Cycloneuralians form a rich and diverse element within Cambrian assemblages of exceptionally preserved fossils. Most resemble priapulid worms whereas other Cycloneuralia (Nematoda, Nematomorpha, Kinorhyncha, Loricifera), well known at the present day, have little or no fossil record. First reports of Sirilorica Peel, 2010 from the lower Cambrian Sirius Passet fauna of North Greenland described a tubular lorica covering the abdomen and part of a well developed introvert with a circlet of 6 grasping denticles near the lorica. The introvert is now known to terminate in a narrow mouth tube, while a conical anal field is also developed. Broad muscular bands between the plates in the lorica indicate that it was capable of movement by rhythmic expansion and contraction of the lorica. *Sirilorica* is regarded as a macrobenthic member of the stem-lineage of the miniaturised, interstitial, present day Loricifera. Like loriciferans, *Sirilorica* is now known to have grown by moulting. Evidence of the life cycle of *Sirilorica* is described, including a large post-larval stage and probably an initial larva similar to that of the middle Cambrian fossil *Orstenoloricusshergoldii* .

## Introduction

Loriciferans are Cycloneuralia, traditionally grouped together with kinorhynchs and priapulids in Scalidophora [1]. The Phylum Loricifera was proposed only 30 years ago [2] and less than 40 of the 100 known species are formally described. All are marine meiobenthic organisms with an adult size range of 0.05 to 0.7 mm. The characteristic longitudinally plated or pleated lorica encloses the abdomen. A transversely folded thorax passes anteriorly via a narrow neck into an introvert carrying numerous scalids arranged in 9 rows with an extended mouth cone. The life cycle of present day loriciferans is both varied and complicated, involving several larval instars before metamorphosis [3,4]. A post-larval stage may precede the adult, with the final larval instar and the post-larva being similar in size to the emergent adult, although larvae which exceed the adult size are known from the deep sea [5]. Growth occurs by moulting and both emerging individuals and abandoned exuviae are well documented [3,6].

Loriciferans are rarely observed alive; usually they are recovered from marine samples which have been shocked with fresh water or from fixed deep sea sediments [5,7,8]. However, the jumping behaviour of adult loriciferans was filmed recently at Roscoff, France [9]. Statements on their ecology are often indirect, such as bacteria are found inside the digestive system or surrounding the mouth cone. The only long-term ecological research on loriciferans is from the hypersaline basins in the Mediterranean Sea where three species of deep sea loriciferans are the only Metazoa living in permanent anoxic sediments [10].

**Figure 1:**
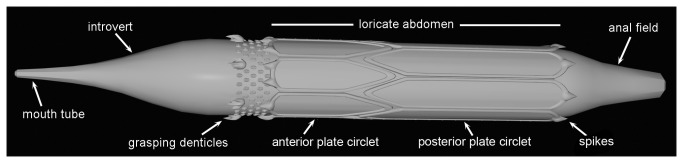
Reconstruction of *Sirilorica carlsbergi* Peel, 2010. Lateral view showing main morphological features, approximately ×1.5.

**Figure 2:**
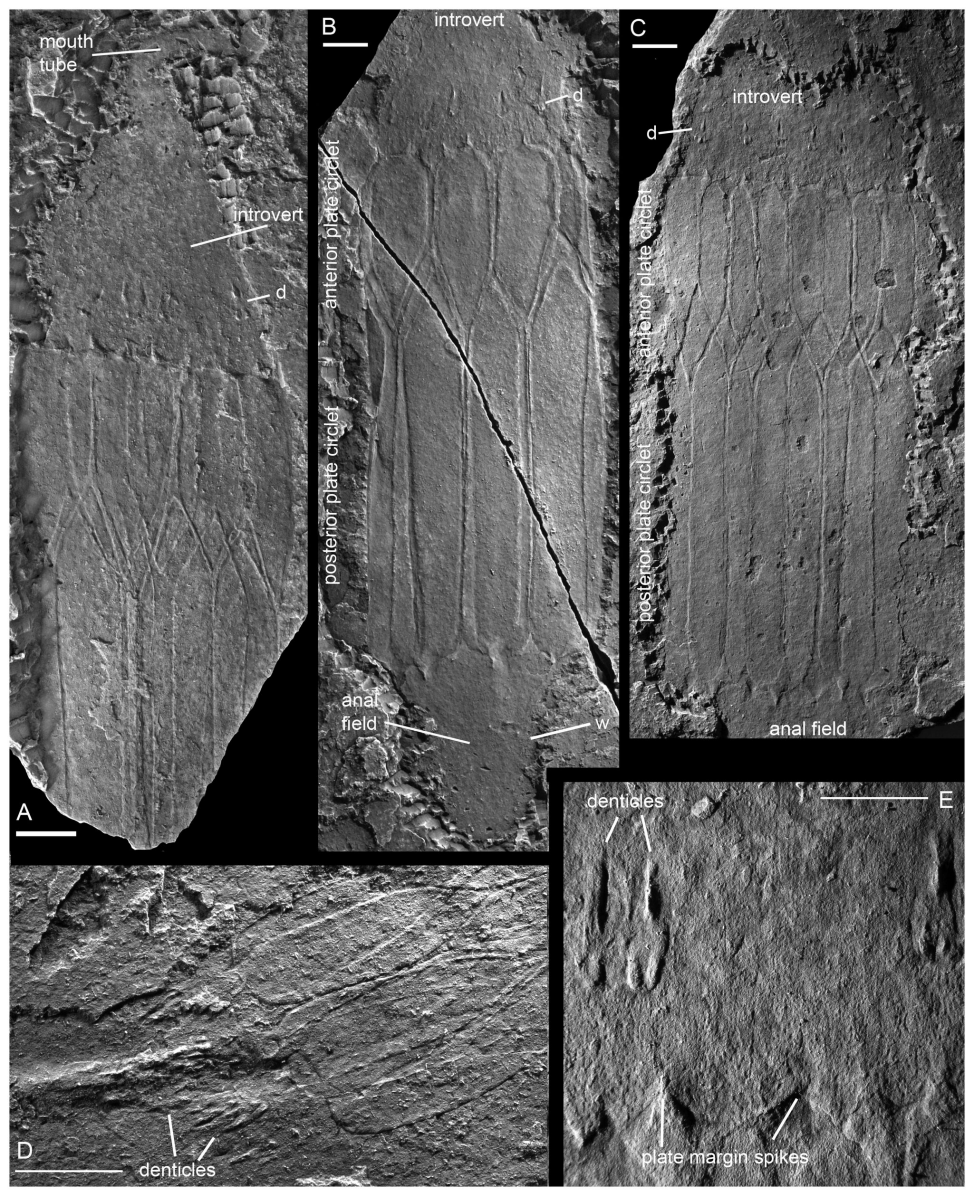
*Sirilorica carlsbergi Peel*, 2010. **A**, lateral view of compressed specimen showing the introvert with mouth tube and the posterior circlet of denticles lying anterior to the lorica. Posterior portion of lorica and anal field not preserved. MGUH 30474. **B**, lateral view of almost complete specimen with conical anal field, see also Figure 4E. MGUH 30475; w, wrinkles parallel to margin of anal field. **C**, lateral view of compressed lorica with denticulate basal portion of introvert, see also Figure 4F,G, 5A. Holotype, MGUH 29155. **D**, anterior margin of compressed lorica with prominent denticles developed on the adjacent introvert. The burrow at the left margin is discussed in the text. MGUH 30476. **E**, detail of ornamented plates with spikes forming the anterior margin of the lorica, showing three large denticles set within the textured introvert. Paratype, MGUH 29156. d, denticles. Scale bars: 4 mm (2 mm in E).

**Figure 3:**
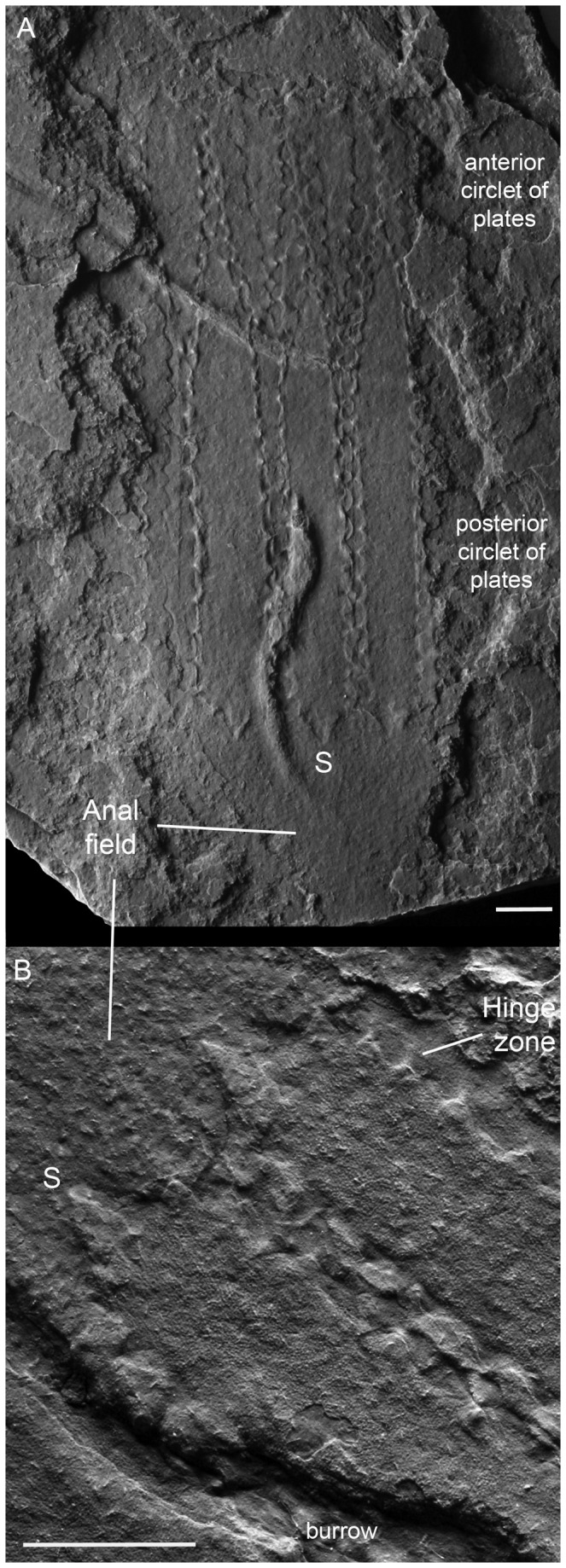
*Sirilorica pustulosa* Peel, 2010. **A**, lateral view of compressed specimen, showing the characteristic lorica with pustulose plate margins, and the conical anal field. MGUH 30477. Two raised burrows cross the lorica. **B**, enlarged detail of the posterior margin of the counterpart of the same specimen showing the prominent single marginal spike on each plate, plate ornamentation and the characteristic striation in the hinge zone visible on the internal surface of an underlying plate. For orientation, the same posterior spike (S) is identified in each figure. Scale bars: 4 mm.

Recently two species of lorica-bearing macrofossils were described from the early Cambrian (Cambrian Series 2, stage 3) Sirius Passet fauna (about 518 Ma) of Peary Land, North Greenland (latitude 82^°^47.6´ N, longitude 42 ^°^ 13.33´W). Despite being more than a hundred times larger than extant loriciferans, they were interpreted as members of the total-group Loricifera [11,12]. *Siriloricacarlsbergi* Peel, 2010 is a common element of the Sirius Passet fauna (Figures 1, 2); its lorica has two circlets each of seven plates similar to the lorica plates of nanaloricid loriciferans [11]. *Sirilorica* *pustulosa* Peel, 2010 [12] is rare but it is readily distinguished from *S* *. carlsbergi* by the pustules along the plate margins (Figure 3). Their first descriptions gave only a brief report on introvert morphology in *Sirilorica* [11] but six large tooth-like structures (denticles) just in front of the lorica resemble the six oral styles of adult nanaloricid loriciferans [13] or the six oral teeth of larval pliciloricid loriciferans [14,15].

Here we describe the anal field, distal details of the introvert, including the mouth tube, and features of internal musculature of *Sirilorica*, allowing reconstruction of the animal in life (Figure 1). The recognition of moulting and post-larvae in the fossil material promotes interpretation of the life cycle of *Sirilorica* by direct comparison with extant Loricifera.

## Materials and Methods

More than a hundred specimens of *Sirilorica* were available from collections made during expeditions to Sirius Passet in 1989, 1991, 1994 and 2006 [11,16] under the auspices of the Geological Survey of Greenland (now a part of the Geological Survey of Denmark and Greenland, Copenhagen, Denmark). Almost all of these specimens, and additional material collected by expeditions organised by the Natural History Museum of Denmark, Copenhagen University, Copenhagen, Denmark, during 2009 and 2011, were collected from locality 1 of the Transitional Buen Formation [17,18], but *Siriloricacarlsbergi* also occurs at locality 2 [18].

All fossil specimens are crushed in shale and were prepared using standard palaeontological mechanical techniques. Subsequently, they were blackened with colloidal carbon and coated with ammonium chloride sublimate from a hot tube prior to photography. The reconstruction (Figure 1) was created in Cheetah 3D, using length-width ratios of the lorica plates from the holotype (Figure 2c). Reconstructing the circle of plates with this ratio allowed determination of the original length-width ratio of the lorica.

All collections, together with type and illustrated specimens (specimen numbers MGUH 30474-MGUH 30484) form a donation to the Natural History Museum of Denmark, and are stored in its premises at Øster Voldgade 5-7, DK-1350 Copenhagen K, Denmark. Access to these collections in connection with this study and publication is approved by the Natural History Museum of Denmark. The Sirius Passet locality lies within the North-East Greenland National Park and all access requires permission from The Greenland Home Rule, Expedition Office, Section of Nature, Post box 1614, 3900 Nuuk, Greenland (exp@nanoq.gl) All necessary permits were obtained for the described study, which complied with all relevant regulations.

### Morphology of *Sirilorica*

Four body regions are recognized in *Siriloricacarlsbergi* (Figures 1, 2): mouth tube, introvert, loricate abdomen, anal field. The anterior narrow mouth tube widens gradually into a conical introvert. The posterior part of the introvert carries a circlet of six large denticles set in distinctly textured cuticle, adjacent to the abdomen. The abdomen is armoured by a cylindrical lorica of robust plates which is preserved in all specimens. A conical anal field forms the posterior termination. In *S* . *pustulosa* only the posterior portion of the introvert, the loricate abdomen and the anal field are known (Figure 3).

The mouth tube is well constrained in a single specimen, showing considerable postmortem flexibility (Figure 2A). In a second specimen (Figure 2D) a broad ridge extends forward across the thorax with the large denticles partly clustered around it. This structure most likely represents a burrow affected by early diagenetic mineralization of a type which is often conspicuous in Sirius Passet fossils [11,19], although a causal relationship between the burrow and the mouth tube is not unlikely. Discernible ornamentation or other morphological structures have not been recognized on the mouth tube or anterior part of the thorax.

**Figure 4:**
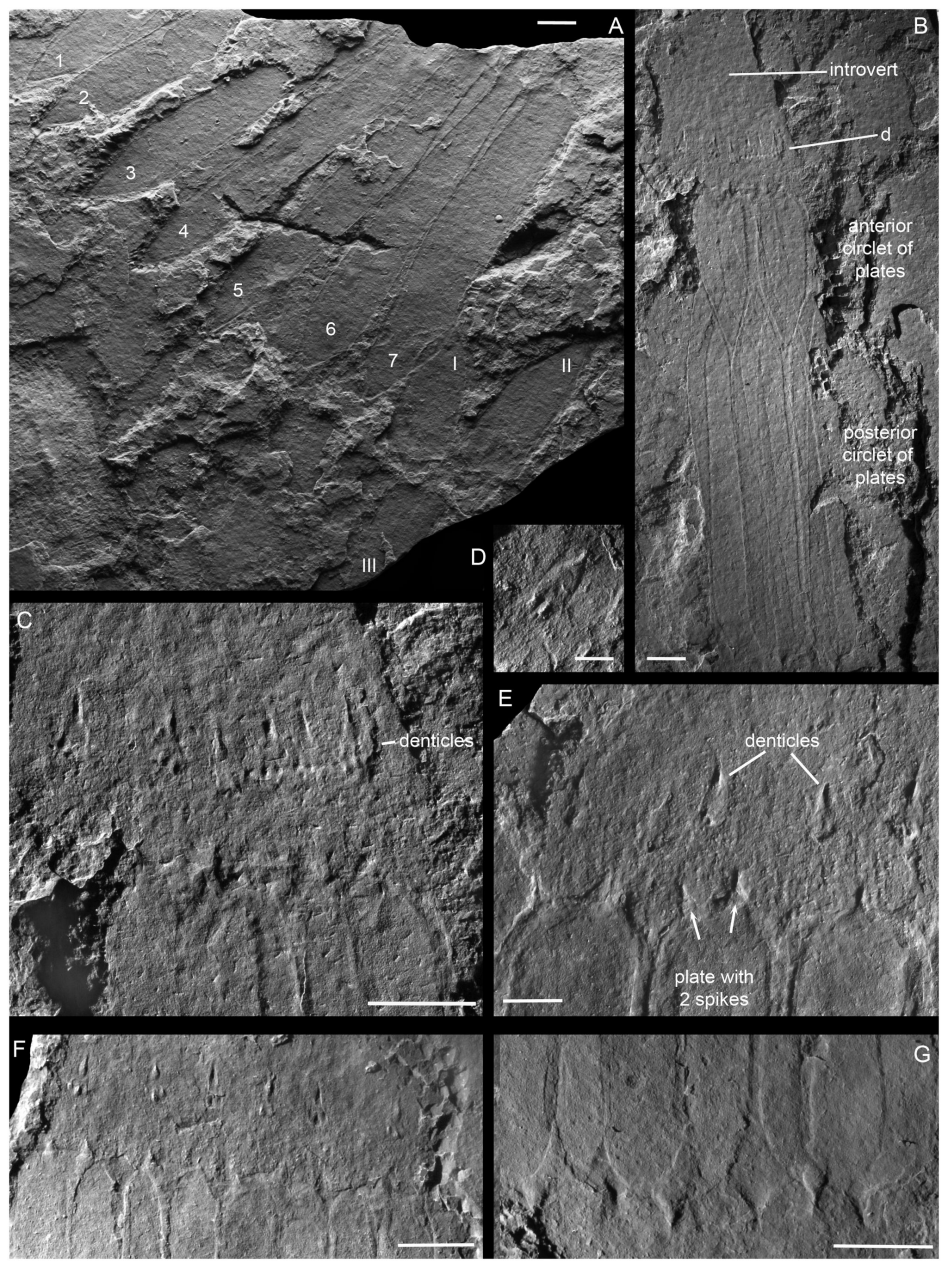
*Sirilorica carlsbergi* Peel, 2010. **A**, disarticulated specimen in which the seven long posterior plates (numbered 1-7 at their anterior, pointed, end) are spread fan-like on the sediment surface. Two of three short, anterior, plates (I, II) show a similar orientation but the third (III) is flipped over such that its pointed posterior margin is now located anteriorly. MGUH 30478, scale bar: 4 mm. **B**, **C**, compressed specimen showing the introvert and circlet of denticles (d) anterior to the lorica, enlarged in C. MGUH 30479, scale bars: 4 mm. **D**, denticle with two posterior cusps and claw-like anterior cusp. MGUH 30480, scale bar: 1 mm. **E**, junction between spike-bearing lorica plates and textured introvert with large denticles, see also Figure 2D. MGUH 30475, scale bar: 4 mm. **F**, junction between spike-bearing lorica plates and textured introvert with large denticles, see also Figure 2C. Holotype, MGUH 29155, scale bar: 3 mm. **G**, posterior margin of lorica with each plate carrying a single spike, see also Figure 2C. Holotype MGUH 29155, scale bar: 3 mm.

The margins of the conical introvert (Figure 2A) are rarely preserved. The most conspicuous feature is the single circlet of large denticles (Figures 2A–E, 4B–G) which is preserved in about 20% of known specimens. The denticles typically occur at a distance from the anterior margin of the lorica approximately equal to their own length (Figures 2E, 4C,E,F) or one fourth the length of the anterior plates of the lorica. The denticles are multicuspidate with two or three shorter basal cusps and a longer central cusp, the tips of which appear to turn slightly inwards towards the axis of the introvert. The margins of the plate carrying the denticles are usually obscure (but see Figure 4D), suggesting that they were overlapped by the surrounding finely tuberculate tissue forming the outer surface of the introvert. The maximum number of denticles observed in any of the studied specimens is six (Figures 2C, 4C,F), though it is frequently less on account of incomplete preservation. It has not been possible to confirm the existence of a seventh denticle within the available material as otherwise might be expected from the presence of seven plates in each circlet of the lorica. The denticles are preserved in positive and negative relief, indicating their original position on the introvert relative to the sediment. The posterior part of the introvert is densely tuberculate. The tuberculate area is often textured longitudinally and this texturing may flow around the denticles (Figures 2E, 4C). Individual tubercles vary in size, with the larger tubercles often located nearer to the lorica; small patches of larger tubercles may indicate several rows of tuberculate platelets, interpreted as microscalids, between the lorica margin and the large denticles, but the margins of such platelets are often obscure, as is also often the case with the larger denticles.

The lorica consists of two circlets, each of seven plates, interdigitating at a transverse zig-zag suture (Figure 2B,C). Plates in the anterior circlet are shorter than those in the posterior circlet, the former comprising about one third of the length of the lorica in the paratype, somewhat more in the holotype. While the plates are now flattened, their juxtaposition indicates that they were curved transverse to their length during life, forming segments within the circular cross-section of the lorica. The plates are rigid, usually with a raised marginal zone, and hinged with adjacent plates along a seemingly flexible hinge. The plates are ornamented with a fine papillation (Figure 2E) which may become linear at the hinge zones; no indication of perforations or other structures penetrating the plates has been observed. The plate margins in some specimens may show a subdued beaded character but this is insignificant when compared to the pronounced pustules developed in *S* . *pustulosa* (Figure 3).

**Figure 5:**
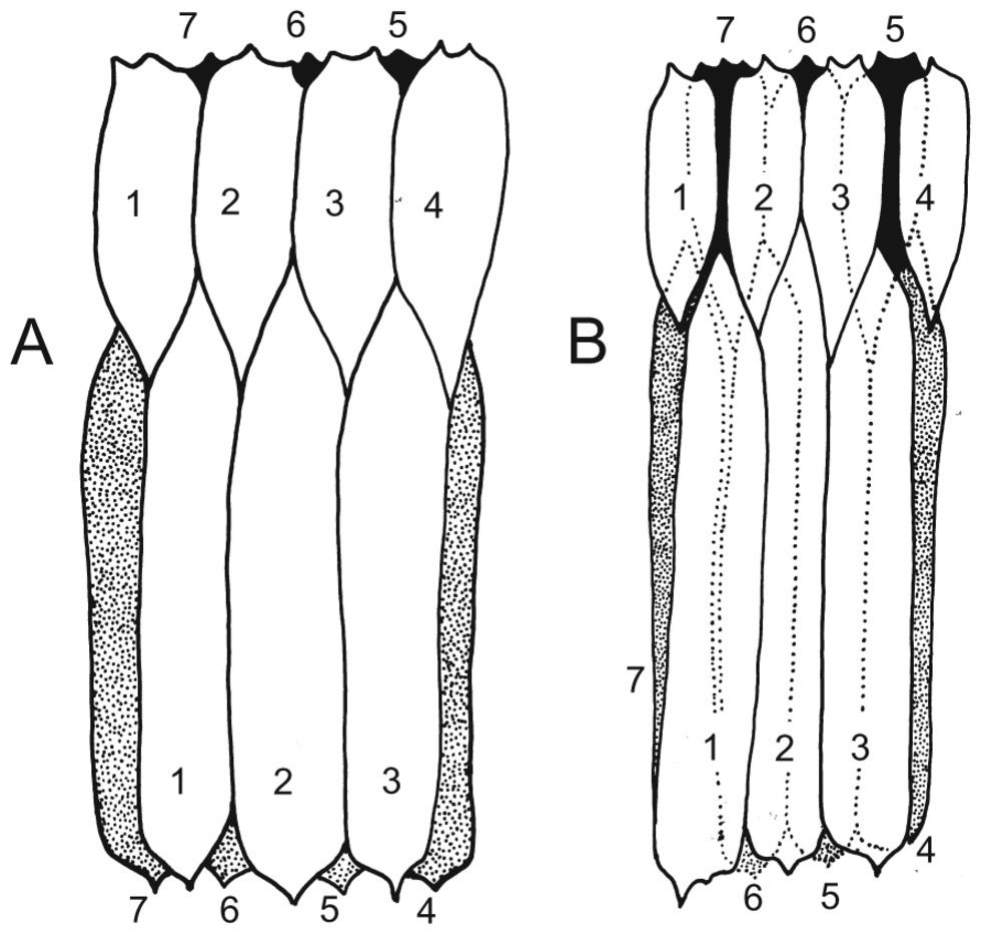
The lorica in *Sirilorica carlsbergi* Peel, 2010. **A**, sketch of holotype showing plates forming the upper surface (unshaded) and lower surface (shaded) of the compacted cylindrical lorica; plates are arbitrarily numbered in an anticlockwise sequence, see also Figure 2C. MGUH 29155. **B**, same, paratype. MGUH 29156.

The lorica in the holotype of *S* *. carlsbergi* (Figure 2C) is 49 mm long; it was originally a cylinder 11 mm in diameter, with individual plates about 5 mm wide [11]. The smallest observed loricae are about two thirds of this length, while fragmentary fossils suggest a maximum length of almost 70 mm. All observed specimens are preserved flattened perpendicular to the longitudinal axis. Collapse on decay and compaction produces a complex pattern as the plate margins from the undersurface are impressed into the overlying plates. Compressions usually show three or four plates on the upper surface overlying, respectively, four or three plates on the undersurface [Figure 5], but in some specimens additional inward folding of the lateral plates produces a concertina-like collapse and what appear to be narrower loricae.

All plates within the lorica carry spikes at their distal margin (anterior for the short plates, posterior for the long plates). Single, double, or triple spikes are present at the anterior margin of the short plates (Figures 2E, 4E,F). Single and double spikes are thickened at their broad bases, bulging away from the plate surface (Figure 2E), and the tips may be extended, claw-like. The middle spike of the triplets is similar to the single and double spikes, but the flanking pair appears to lack the basal thickening (Figure 4F). Characteristically the spikes are preserved in greater relief than the rest of the plate, suggesting that they were conical in form and originally more robustly sclerotized. While the variation in number of spikes disturbs the radial symmetry of the plates of the lorica it has not been possible to identify a clear and consistent plane of bilateral symmetry on the basis of the distribution of spikes. The plates of the posterior circle have a single spike each (Figure 4G). These spikes seem to be better defined than the anterior spikes, and they may have a more oval base.

**Figure 6:**
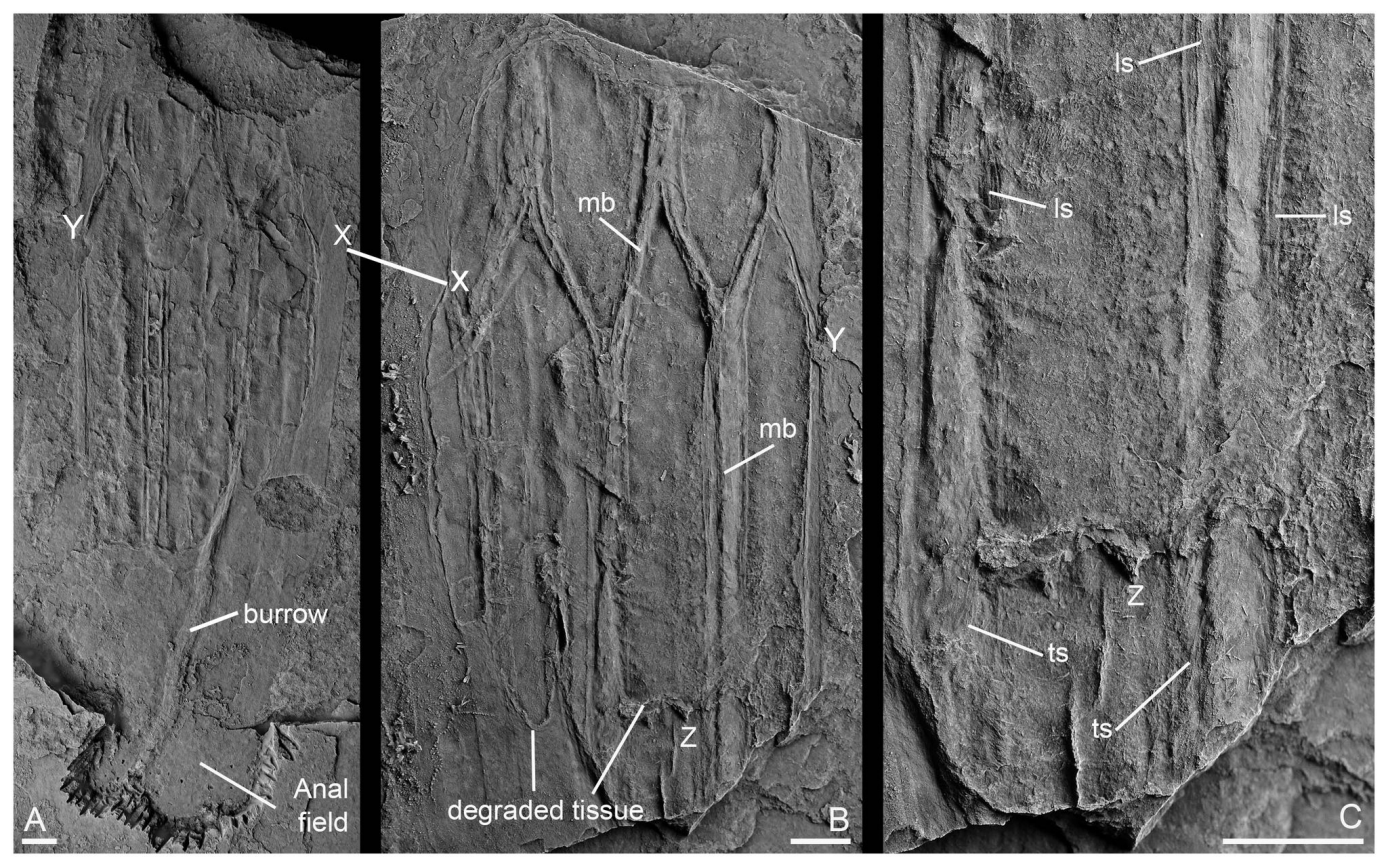
Internal structures within the lorica of *Sirilorica carlsbergi* Peel, 2010. **A**, compressed specimen showing the internal surface of the lorica and spatulose anal field crossed by a burrow. **B**, counterpart of the same specimen with X-X joining equivalent points of the two originally opposing surfaces which are now placed side by side. Y indicates equivalent points in A and B. Note the broad striated muscular bands (mb) extending from one plate to the next across the intervening hinge zone, and degraded margins of lining tissue. **C**, enlarged detail of B showing the longitudinal (ls) and transverse, crescentic, striations (ts) of muscle fibres in the muscular bands; Z indicates equivalent points in B and C. MGUH 30481, scale bars: 3.5 mm.

The conical anal field has been observed in only three specimens of *S* *. carlsbergi* (Figures 2B, 6A) and one of *S* *. pustulosa* (Figure 3A). There is no discernible trace of morphological structures other than a papillate texture (Figure 3A) and a slight compactional wrinkling adjacent and parallel to the outer margins (Figure 2B). It is unclear if the papillate texture is an artifact of diagenetic recrystallization.

A single specimen preserved as part and counterpart (Figure 6) is unusual in that breakage seems to have occurred between the layers of plates forming the upper and lower surfaces, respectively, of the collapsed lorica; the interior surface of the plates is thus visible. Of particular note are well preserved, broad, longitudinal muscle bands overlapping the plate junctions and occupying a much greater width than the hinge zones when viewed externally. Some of the raised character of plate margins when viewed from the exterior may reflect the impression of this muscular hinge zone through the plates. The muscle bands show fine longitudinal and transverse striation (Figure 6C). Similar cases of muscle preservation have been reported previously from Sirius Passet [20–23]. The nature of this broad zone of muscular attachment between the plates indicates that the plates could move relative to each other along their margins. *Sirilorica* was capable of zonal expansion and contraction of the lorica, in terms of its diameter, thus providing a mechanism for locomotion.

Large areas of the internal surface of the lorica are draped with a thin, partly degraded, sheet of tissue or decomposition product of the epidermis (Figure 6B,C). This sheet may be wrinkled but is otherwise structureless. No other traces of internal structure have been recognized in the available material.

### Life cycle and growth of *Sirilorica* and Loricifera

Present day loriciferans have complicated and variable sexual/asexual life cycles [3,4,7,24]. A relatively simple sexual life cycle in nanaloricid loriciferans involves 2–5 larval instars being present before metamorphosis, while a post-larval stage very similar to the female may precede the adult [2,15,25]. The final larval instar and the post-larva may be of similar size to the emergent adult. Growth occurs by moulting and the occurrence of emerging individuals and abandoned exuviae is well documented [3,6]. Both the empty exuviae of the larvae with toes (Higgins larvae) and the post-larva, always without toes, are frequently observed in sediments [6,14].

**Figure 7:**
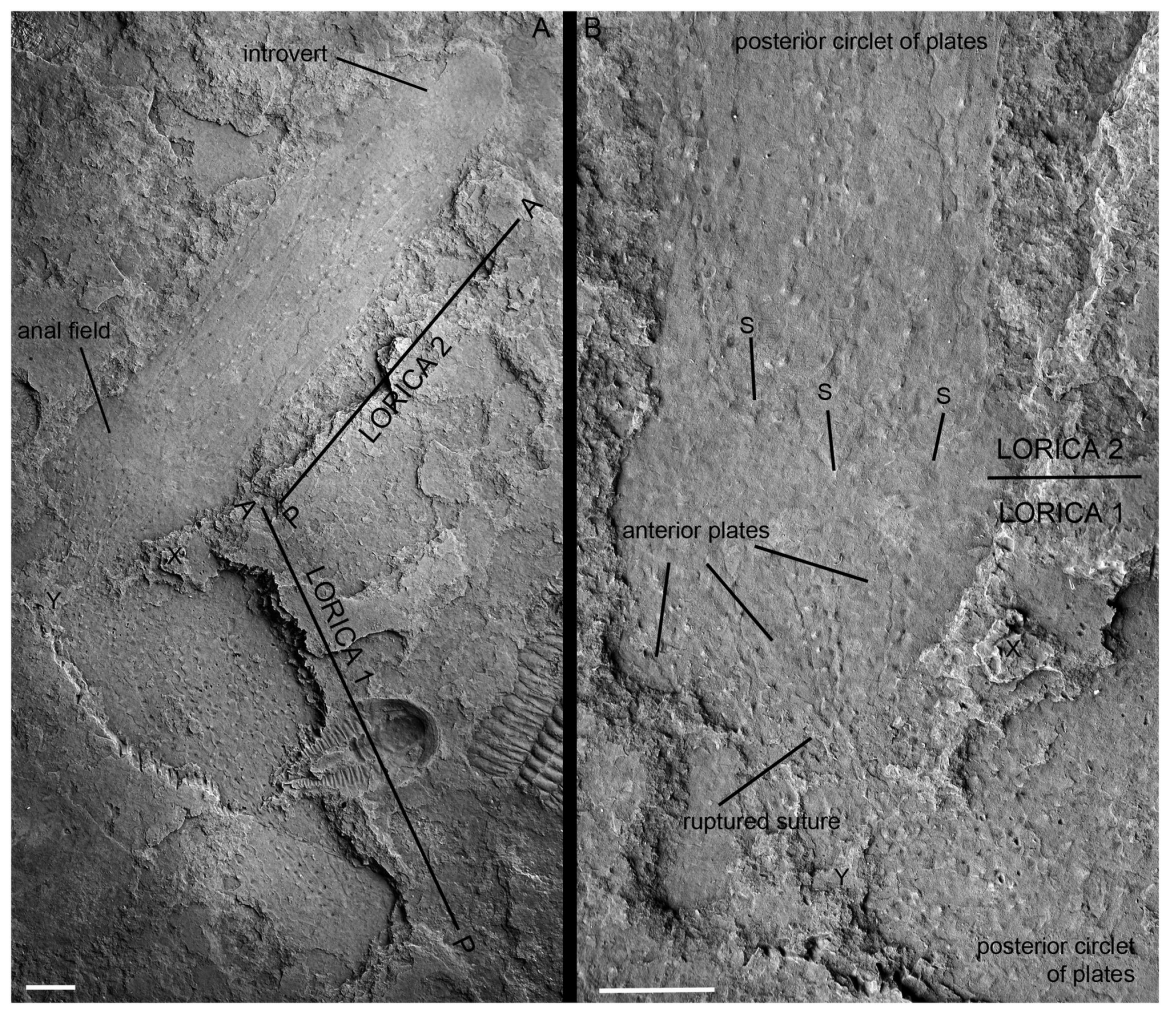
Moulting in *Sirilorica pustulosa* Peel, 2010. **A**, compressed specimen with introvert and anal field (Lorica 2) recently emerged from the exuvia (Lorica 1). **B**, enlargement of A showing rupturing of the suture separating the anterior and posterior circlets of plates in the lorica of the exuvia (lorica 1), with anterior plates splaying away from the rupture. X and Y indicate equivalent points in A and B. S, spikes on the posterior plate margins of lorica 2. MGUH 30482. Scale bars: 5 mm.

Direct evidence of moulting in *Sirilorica* *pustulosa* is provided by a single, nearly complete specimen that is closely associated with a deformed lorica of the same species (Figure 7). The anterior specimen, interpreted as the emergent individual, preserves part of the thorax with traces of denticles and the lorica with its characteristic two circlets of plates with pustulose margins (Figure 7, lorica 2). Only the portion of the anal field directly adjacent to the abdomen is visible; it bulges laterally beyond the width of the lorica, then rapidly narrows where it is braced by the plates of the anterior circlet of the posterior specimen, interpreted as the exuvia (Figure 7, lorica 1). The plates of the anterior circlet of the exuvial lorica are splayed in a manner not known from other specimens of *Sirilorica*. As a result of this anterior splay, the plates converge strongly posteriorly towards the suture with the longer plates of the posterior circlet of lorica plates which are angulated away from the ruptured or distorted suture, forming a strong constriction there. No introvert of the exuvia can be observed.

Moulting from post-larva to adult in Recent loriciferans is well known from species of *Pliciloricus* and *Rugiloricus*; in a moulting specimen of *Rugiloricus* *ornatus* the adult was seen to be released from the anterior part of the post-larva [14], exactly as in the fossil described herein. An alternative explanation of the juxtaposition of the two fossil specimens could be cannibalism, which has been observed in Recent and Cambrian priapulids [26,27], but the lack of ingestion, the orientation of the specimens, the distorted and ruptured lorica and the absence of an introvert in the posterior specimen render this unlikely.

One specimen of *S* *. carlsbergi* has been completely split open such that all 7 of the longer, posterior plates are lying next to each other on the sediment surface (Figure 4A). Three short, anterior plates are preserved but one of these (Figure 4A, plate III) has been inverted. Given the degree of demolition when compared to the exuvia described above (Figure 7, lorica 1) it is likely that the specimen represents a predated *Sirilorica*.

**Figure 8:**
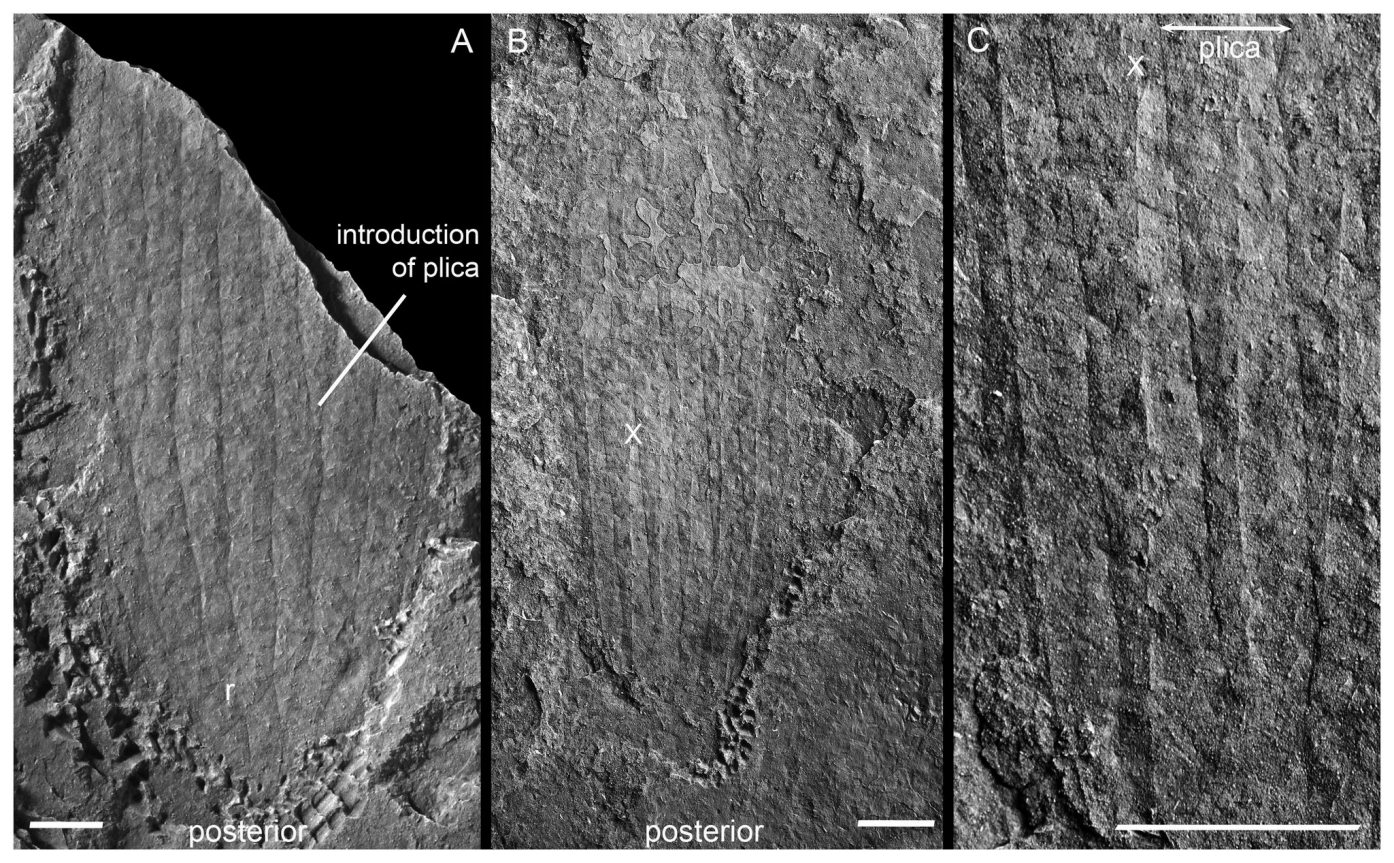
Post-larva of *Sirilorica* Peel 2010. **A**, lateral view of compressed lorica showing the posterior convergence of plicae; r indicates rectangular structure discussed in the text. MGUH 30483. **B**, as A. MGUH 30484. **C**, enlargement of B (X indicates equivalent points) showing the characteristic valley-and-ridge structure of the plicae and finely pustulose ornamentation. Scale bars: 3 mm.

Two smaller loricae (Figure 8A,B) are interpreted as larval stages of *Siriloricacarlsbergi* . It cannot be conclusively ruled out that these may represent a different cycloneuralian taxon. However, their general form, the presence of moulting individuals (described above) and comparison with the known life cycle of extant loriciferans promote the interpretation as larvae or post-larval exuvia. The plicated loricae look similar to those of the adults of Recent *Pliciloricus* and *Rugiloricus* [25,28] but the two fossil specimens are several hundred times larger than larvae of Loricifera and Priapulida. The most complete of the two flattened specimens (Figure 8B) has a preserved length of 38 mm and width of 11 mm, amounting to about the same size as the smallest known undoubted specimens of *Siriloricacarlsbergi* and half the length of the largest ones. The preserved width corresponds to a diameter of between 7 and 11 mm, depending on the means of compaction of the lorica; its lateral margins and both terminations are not preserved, but there is a distinct initial expansion from one termination (designated as posterior for purposes of description) after which the lorica quickly becomes parallel-sided anteriorly. The second specimen (Figure 8A) is wider (17.5 mm) and thus originally was probably longer. While its lateral and distal margins are incomplete, the proximal (posterior) area is quite well preserved.

Unlike specimens confidently assigned to *Sirilorica*, where plates from the underside are impressed through those on the upper surface of the fossils (Figure 2C), the lorica of the larvae lacks separate plates and only one side of the lorica is visible when crushed. On this visible side, the lorica is divided longitudinally into a series of subparallel-sided plicae whose folded surfaces, produce the pleated structure (Figure 8C). During the first quarter of its preserved length, the lorica expands slightly as new plicae are inserted. At the maximum width (uppermost, anterior, in Figure 8B) some 7 to 8 plicae can be counted, so that there are up to 16 plicae in total. For comparison, the the morphologically similar lorica of *Pliciloriusenigmaticus* [28] and the larvae of most pliciloricid loriciferans [14 but see 5] have up to 30 plicae. The plicae margins show incipient pustulosity (Figure 8C), as sometimes seen at the plate margins in *Siriloricacarlsbergi* , in contrast to the prominent pustulose margins of *S* *. pustulosa* . The pleated structure is less well developed in the other larva (Figure 8A). Ten plicae are preserved anteriorly; this number is achieved by the interdigitation of plicae, most clearly seen at about half the preserved length (ca 16 mm) where two plicae are introduced, initially accompanied by a relative narrowing of adjacent plicae. The posterior convergence of plicae strongly suggests a single point of origin from which a small number of plicae diverge; at least three plicae are visible on the flattened surface at this stage. Two (possibly four) new plicae are seen to have been introduced on the flattened surface already after 5 mm. The posterior area also preserves a regular, rectangular, structure (1.5 mm x 3.5 mm; Figure 8A, r) which lies oblique to the plica boundaries and overlies them in the specimen as preserved. It has the same width and texture as the longitudinal plates, suggesting it could be a detached fragment, although its regular form may indicate that it represents a detached, but separate skeletal element or that it is completely extraneous. Very fine striations are parallel or slightly oblique to the length of the plates (Figure 8A); the fine papillation visible in patches on both specimens seems largely to reflect diagenetic recrystallisation. No evidence of structures penetrating the walls of the lorica has been observed.

Evidence of other growth stages is not yet available for *Sirilorica*. However, the pattern of pleated plicae on the lorica is reminiscent of that seen in larvae assigned to the species *Orstenoloricusshergoldii* Maas, Waloszek, Haug & Müller, 2009 from the middle Cambrian (Cambrian Series 3) of Queensland, Australia [29]. The specimens were recovered by digestion of carbonate rocks by weak acids, a technique which is not applicable to the siliciclastic sediments that yield the Sirius Passet fauna [17]. They are less than 1000 µm in total length (similar in size to the largest Recent loriciferan larvae), compared to the more than 38 mm of the supposed larval loricae from Sirius Passet. Some 20 plicae are described in the larvae of *O*. *shergoldii*, converging towards the inferred posterior, but evidence of the introduction of additional plicae into this series was not described. Outgrowths in the anterior region of the lorica and in one specimen also in the posterior region of the lorica are interpreted as the basal part of the sensory setae found in recent Higgins larva of loriciferans [3,4,14]. This may support the interpretation of the larvae of *Orstenoloricusshergoldii* as fossil loriciferan larva.

### *Sirilorica* as a stem-group loriciferan

Loriciferans, priapulids (together forming Vinctiplicata) and kinorhynchs have been considered to comprise Scalidophora within Cycloneuralia [1,30]. The autapomorphic character of Vinctiplicata is the development of the lorica yet the nature of the lorica varies considerably within loriciferans and priapulids. All loriciferans have a lorica, but only nanoloricerid Loricifera have lorical plates with spikes (as does *Sirilorica*) [2–9]. Adult priapulids lack a lorica but various plated or pleated loricae are present in larvae of the few Recent species, although one has direct development and lacks a larval lorica [31–34]. Several rotifers (Lophotrochozoa, Gnathifera) also develop a non-moulting larval lorica indicating convergent devolpment of the lorica within the meiofauna. Indeed, the plated and spiked lorica of the rotifer *Notholcaikaitophila* Sørensen & Kristensen, 2000 shows a remarkable resemblance to *Sirilorica* [35]. This morphological variation is increased with the inclusion of fossil forms and the notion that the lorica may not be a unifying character for Vinctiplicata but was developed on at least two occasions with Cycloneuralia has gained support through recent molecular studies [30,31]. These have questioned the validity of Scalidophora, instead supporting a sister-group relationship between Loricifera and Nematomorpha [36,37]. While morphological similarities between marine nematomorph larvae and the adult loriciferan *Nanaloricusmysticus* add support to this interpretation [2,11], the proposed relationship between Loricifera and Nematomorpha is not in accord with traditional morphologically based phylogenetic models [1] and has been rejected by others [29,30,38]. However; new ultra-structural research [4] demonstrates that also the buccal tube retractors of loriciferans have a hexaradiate symmetry like the anterior muscles in nematomorphs [9]. Six large grasping denticles are also present on the introvert of *Sirilorica* (Figures 2C, 4F).

The association of Kinorhyncha, Loricifera and Priapulida within Scalidophora [39] fostered the hypothesis that scalidophorans are plesiomorphically metameric, based largely on the inferred phylogenetic position of *Markuelia* Val’kov, 1983 in the scalidophoran stem-group [39–41] or total-group [38]; the latter reflecting uncertainty as to its placement as either a stem-group priapulid or stem-group scalidophoran. Evidence of metamerism is always present in Kinorhyncha, with 11 trunk segments [42], but is lacking in other extant Scalidophora and in *Sirilorica*.

Fossil Cycloneuralia are common in the Cambrian. While many are discussed as representatives of the priapulid stem or crown groups [30,38,39,43–47] their true status within Cycloneuralia is unresolved [30]. Kinorhynchs remain unknown as fossils. Extant loriciferans are microscopic but their morphological complexity indicates that they developed from much larger animals [3]. Huang Diying (2005, unpublished Ph.D thesis, University Claude Bernard, Lyon 1; see 48) claimed that some of the so-called priapulid larvae from the lower Cambrian in China (such as *Sicyophorus* *rara* Luo & Hu *in* Luo, Hu, Chen, Zhang & Tao, 1999) are adult loriciferans [49]. He also pointed out that some specimens of *Sicyophorus* *rara* have anterior spikes on the lorica like nanaloricid loriciferans and *Sirilorica*. *Sicyophorus* has been interpreted as a derivative of the loriciferan stem-lineage largely on account of the urn-like lorica [45], an argument which gains validity in the present context, but it has also been placed within the priapulid total-group [11,38,39].

*Sicyophorus* *rara* may be locally abundant within the Chengjiang lagerstätte and specimens have a total length, inclusive of lorica and introvert, of 5–10 mm. This is 10 to 20 times larger than extant loriciferans, but its pleated lorica is also 20 times smaller than that of *Sirilorica*. Little evidence of the prominent multiple spinosity characteristic of the introvert of *Sicyophorus* *rara* is seen in *Sirilorica*. Its lorica is urn-shaped, closed posteriorly, unlike the parallel-sided lorica of adult *Sirilorica*. However, specimens from Sirius Passet interpreted as larval *Sirilorica* are also closed posteriorly (Figure 8) and the nature of their introvert is not known. *Sicyophorus* (as its synonym *Protopriapuliteshaikouensis* Hou, Bergström, Wang, Feng & Chen, 1999) is characterized by a strongly coiled intestine that is otherwise not known within Scalidophora [48,50,51].

A pleated lorica is also preserved in the vase-shaped lorica (length 5 mm) of a specimen referred to as an un-named paleopriapulitid from the Kaili Biota (Middle Cambrian) of Guizhou [52]. About 20 plates are visible in lateral view but details of plate insertion have not been recognised. The claviform introvert contrasts with the conical introvert of *Sirilorica* and carries several rows of posteriorly directed spines.

The loricate *Orstenoloricusshergoldii* [29] ranges in size from 0.25 mm to 0.5 mm, the same size as the largest Recent loriciferans. Outgrowths in the anterior region of the lorica and in one specimen also in the posterior region of the lorica seem to represent the basal part of the sensory setae found in recent Higgins larva of loriciferans [3,14], supporting interpretation of *Orstenoloricusshergoldii* as a fossil loriciferan larva. A second species from the Cambrian “Orsten” fauna from Queensland, *Shergoldana* *australiensis* Maas, Waloszek, Haug & Müller, 2007, has been described to accommodate a possible larval roundworm [46] which may be related to priapulid larva or adult kinorhynchs. Alternatively this tiny (0.145 mm) larva may be the larva of a palaeoscolecid worm [41].

The complexity of present day loriciferans, witnessed not least by the numerous circlets of more than 300 scalids on the introvert and neck [53], indicates that they were derived from much larger ancestors [3]. The macrobenthic species of *Sirilorica* are several hundred times larger than present day loriciferans but their known morphological organisation is relatively simple by comparison; the complexity of the miniaturised present day loriciferans no doubt reflects specialisation in their interstitial habitat. In gross morphological terms, *Sirilorica* resembles present day loriciferans in terms of its well-developed lorica and introvert with an extended mouth tube. The adult lorica is, however, open at both ends, unlike the posteriorly closed lorica of present loriciferans, although the putative post-larval lorica of *Siriloricacarlsbergi* is closed posteriorly (Figure 8). The introvert of *Sirilorica* carries one circlet of 6 prominent denticles, although its textured surface suggests the presence of several rows of tubercles or miniscalids. Unlike the scalids of loriciferans (and priapulids) these denticles are curved inwards with a clear grasping function [11], suggesting that their equivalence lies more with the hexaradial oral teeth or valves of present day loriciferans [7,14,54] rather than with the numerous scalids of the introvert [15,25,28].

The ground pattern of the mouth cone in all loriciferans is the hexaradial arrangement of the buccal tube [15], oral styles/oral valves in both in larvae and adults [13,14] and a triradial myoepitheal pharyngeal bulb with placoids (cuticular thickenings formed by myoepitheal cells). Furthermore, the internal armature (buccal armature) in all *Pliciloricus* larvae is hexagonally arranged [54]. Only 4 oral styles/valves are present in a few species of *Rugiloricus* and some *Nanaloricus* species lack outer oral styles. However, oral styles or internal armature that are always formed as a hexaradial star with teeth or styles are present inside the mouth cone of all adults, as well as larvae of extant loriciferans [54].

The longitudinal and transverse, crescentic muscles in *Sirilorica* (Figure 6, ls and ts) are similar to the abdominal longitudinal muscles [9] and to the lorical plate muscles ([28] fig. 1) in nanaloricid loriciferans. The lorical plate muscles in *Nanaloricus* are arranged in four clusters ([9] fig. 3) which may be attached to the six plates in several different ways ([13] fig. 13,dv). The presence of tranverse muscles attached to the hinge zone of the plates in both *Sirilorica* and *Nanaloricus* and a hexaradial pattern in the mouth tube/mouth cone suggest that these characters already existed in the stem-lineage of Loricifera.

The anatomical ground pattern of Loricifera as a total-group, including both the macrofaunal extinct *Sirilorica* and the meiofaunal extant Loricifera, thus comprises: 1) a retractable mouth tube/mouth cone with a hexaradial pattern of denticles/oral styles; 2) an eversible introvert with scalids; 3) a loricate abdomen with spiked plates; 4) a naked anal field/anal cone with the anus.

The proposed sister-group relationship between Loricifera and Nematomorpha promotes the suggestion that loriciferans evolved through progenesis, with sexual maturation of the lorica-bearing larva in a worm-like ancestral form [37]. The same mechanism can be inferred for the evolution of present day loriciferans from *Sirilorica*, with loss of the tubular lorica and anal field accompanying miniaturisation with retention in the adult of an urn-shaped lorica comparable to the post-larvae of *Sirilorica*. The Chinese *Sicyophorus* , with its urn-shaped lorica, may represent a morphologically intermediate case within the stem-lineage of Loricifera [44] or a parallel development within priapulids if its affinities have been correctly interpreted [38,39,47].
